# Compound Evolutionary History of the Rhesus Macaque *Mhc* Class I *B* Region Revealed by Microsatellite Analysis and Localization of Retroviral Sequences

**DOI:** 10.1371/journal.pone.0004287

**Published:** 2009-01-27

**Authors:** Gaby G. M. Doxiadis, Corrine M. C. Heijmans, Maxime Bonhomme, Nel Otting, Brigitte Crouau-Roy, Ronald E. Bontrop

**Affiliations:** 1 Department of Comparative Genetics and Refinement, Biomedical Primate Research Centre, Rijswijk, The Netherlands; 2 Department of Evolution and Diversity Biology, University Paul Sabatier, Toulouse, France; Max Planck Institute for Evolutionary Anthropology, Germany

## Abstract

In humans, the single polymorphic *B* locus of the major histocompatibility complex is linked to the microsatellite MIB. In rhesus macaques, however, haplotypes are characterized by the presence of unique combinations of multiple *B* genes, which may display different levels of polymorphism. The aim of the study was to shed light on the evolutionary history of this highly complex region. First, the robustness of the microsatellite MIB-linked to almost half of the *B* genes in rhesus macaques (*Mamu-B*)–for accurate *B* haplotyping was studied. Based on the physical map of an established haplotype comprising 7 MIB loci, each located next to a certain *Mamu*-*B* gene, two MIB loci, MIB1 and MIB6, were investigated in a panel of MHC homozygous monkeys. MIB1 revealed a complex genotyping pattern, whereas MIB6 analysis resulted in the detection of one or no amplicon. Both patterns are specific for a given *B* haplotype, show Mendelian segregation, and even allow a more precise haplotype definition than do traditional typing methods. Second, a search was performed for retroelements that may have played a role in duplication processes as observed in the macaque *B* region. This resulted in the description of two types of duplicons. One basic unit comprises an expressed *Mamu-B* gene, adjacent to an HERV16 copy closely linked to MIB. The second type of duplicon comprises a *Mamu-B* (pseudo)gene, linked to a truncated HERV16 structure lacking its MIB segment. Such truncation seems to coincide with the loss of *B* gene transcription. Subsequent to the duplication processes, recombination between MIB and *Mamu-B* loci appears to have occurred, resulting in a hyperplastic *B* region. Thus, analysis of MIB in addition to *B* loci allows deciphering of the compound evolutionary history of the class I *B* region in Old World monkeys.

## Introduction

Gene products of the Major Histocompatibility Complex (MHC), a multicopy gene system present in nearly all vertebrate species, play a key role in immune-related defense reactions. MHC class I molecules, for instance, are involved in the binding and presentation of intracellular generated peptides to CD8^+^ T cells, whereas class II molecules present peptides from extracellular origin to CD4^+^ T cells. The hallmark of the *Mhc* class I and II genes is their abundant polymorphism as well as gene copy number variation observed between as well as within species [1]–[4].

Due to its prominent role in disease susceptibility/resistance and transplantation biology, the MHC region has been studied extensively in humans (HLA) and non-human primates [5]–[11]. Equivalents of the classical *HLA* class I genes, *HLA-A* and *HLA-B,* have been defined in the rhesus macaque (*Macaca mulatta*), and are designated *Mamu-A* and *-B* [12]–[15]. The orthologue of the classical *HLA-C* locus, however, is absent in the rhesus macaque. On the other hand, both classical class I genes, *-A* and *-B*, are multiplicated in rhesus macaques and should therefore be considered as paralogues [14], [16], [17]. Equivalents of the nonclassical *HLA* class I genes *HLA-E* and *-F* that are characterized by a low degree of polymorphism and differential tissue distribution, have also be detected in the rhesus macaque and have been named *Mamu-E and -F* [18], [19]. In particular, the ancestral *B* gene seems to have been subject to expansion during the evolution of the rhesus macaque, which started 23–31 million years (Myr) ago [20], as was concluded based on the observation that a particular MHC region comprises multiple *Mamu-B*-like genes [16], [21]. Analysis of a large panel of rhesus macaques of Indian and Chinese origin showed that the copy number and content of *B* loci could vary significantly per chromosome [22]–[24]. Some of these genes, for example *Mamu-I* (also named B3), may represent nonclassicals with specialized functions [25].

In the past, when molecular methods were not yet available, typing has been performed with alloantisera in analogy to the human situation, and 16 Mamu-B specificities (serotypes) have been defined [12]. For each serotype at least one unique combination of transcribed *B* genes has been determined in animals of Indian origin [23]. Apart from qualitative dissimilarities, quantitative differences are also observed. Based on expression levels, rhesus macaque MHC class I gene products can be divided into majors and minors. Serotyping is a complex, expensive, and outdated technology; in addition, class I gene sequencing is highly cumbersome. An easier and thorough way of characterizing and typing the *B* region is to study closely linked markers such as microsatellites. To develop a fast and accurate typing protocol, an *in silicio* search for *Mamu-B* region-associated microsatellites was performed, and D6S2810 (MIB) was selected as a promising candidate. MIB, a (CA)n dinucleotide repeat that is polymorphic in length, shows nucleotide variations, and is closely linked to the *Mhc-B* locus/loci, both in hominoids and Old World Monkeys [26]–[28]. Seven different loci, named MIB1 to 7, could be defined next to certain *Mamu-B* loci on the haplotype that covers the complete *Mamu-B*, and class II and III regions by *in silicio* analysis [16], [29]. These data suggest that the region has been subjected to expansion. Indeed, nineteen *Mamu-B* genes are present on this haplotype, of which all but one of the nine telomerically oriented *B* genes are associated with one MIB copy [16], [29]. The ten other *B* genes are not accompanied by a MIB structure. To assess the robustness of MIB microsatellite typing to infer *B* serotypes and complex *B* haplotypes, MIB1 and MIB6 of homozygous rhesus macaques have been analyzed by genotyping and sequencing.

In order to learn more about the possible mechanisms leading to duplications, a study of the genomic environment of genes, including transposable elements, may be helpful. Retrotransposons, such as ERV, MIR, MLT and LTR, contribute strongly to the diversification of gene families by way of insertions/deletions within intergenic and intragenic duplicated regions or by acting as recombination hotspots [30]–[36]. For example, within the *Mamu-A* region (alpha block), 28 duplicons have been described and HERV16 sequences appear to map directly to the breakpoints [14]. In humans, a long HERV16 sequence has also been observed centromeric of *HLA-B* [33]. Tandem duplications of HLA genes and HERV16 sequences with and without MIC genes have been hypothesized [35]. For these reasons, we intended to screen for such retroelements and for their potential implications in the functional and evolutionary genomics of the duplication processes within the class I *B* region of rhesus macaques.

## Materials and Methods

### Animals

The Biomedical Primate Research Centre houses a self-sustaining outbred colony of about 650 rhesus macaques that have been pedigreed based on the segregation of serologically defined MHC haplotypes. Two unrelated families of Indian origin were selected from this colony. One family consists of one male, five females, and five offspring; additionally, the MHC-identical sibling of one female together with her male and three offspring was analyzed. The other family consists of one male, four females, and 18 offspring. An inbreeding program resulted in a group of Mamu-A, -B, and -DR homozygous animals of consanguineous origin [37]. 21 Unrelated animals of Indian origin were chosen from this group for subsequent studies.

All procedures as drawing blood from theses animals were performed in accordance with the guidelines of the Animal Care and Use Committee installed by Dutch law.

### Serological MHC typing

The animals were serologically typed for MHC class I antigens, and 14 Mamu-A and 16 Mamu-B serotypes were defined. Serotyping was performed by polyclonal sera raised by active immunizations of mainly Indian rhesus macaques [12]. A cluster of positive typing reactions defined serotypes.

### MIB1 and MIB6 genotyping

DNA from immortalized B cells or fresh EDTA blood was isolated using a standard salting-out procedure. For amplification of MIB1 and MIB6 sequences, two specific primer pairs were developed. In both reactions, the same fluorescent forward primer, MIBforward, was used: FAM-GAT TCT TCA GAG AAG CAG AACC, whereas the reverse primer is specific for the MIB1 or MIB6. MIB1 reverse: ATT CTG CCT TTC TGC GTT TT; MIB6 reverse: CTG CAG ATT TTC GTA TGT AC. PCR amplifications were carried out starting with 5 min at 94°C, followed by 5 cycles of 94°C 1 min, 58°C 45s, and 72°C 45s, then followed by 25 cycles of 94°C 45s, 58°C 30s, and 72°C 45s. Followed by a final elongation at 72°C for 30 min. For the PCR, the following products were used: 10× buffer, 2.5 μM MgCl, 0.2 μM of each dNTP, 0.5 U Taq pol. PCR was run on a GeneAmp PCR system 9700 (Applied Biosystems, Foster City, CA). For the fragment analysis, 1 μl op PCR product and 15 μl of HiDi was used (Applied Biosystems). The fragments were run and analyzed on a 3130*XL* automatic analyzer (Applied Biosystems) with Genescan-350 ROX as size standard.. Analysis was performed using the GeneMapper program (Applied Biosystems).

### Cloning and sequencing of MIB amplicons

PCR amplification of MIB1 and MIB6 was performed as described above. PCR fragments were purified using a QIAquick gel extraction kit (QIAgen GmbH, Germany) according to the manufacturer's instructions. PCR products were then cloned into the pDRIVE cloning vector by using the Qiagen PCR cloning kit (QIAgen GmbH, Germany ) in accordance with the manufacturer's instructions. After transformation, colonies were picked and plasmid DNA was isolated using a standard mini-preperation procedure. Purified (plasmid) DNA was sequenced on the ABI 3100 or ABI3130*XL* automatic analyzer (Applied Biosystems) by using 0.2 μM M13 primer, 1 μl BigDye, and 2 μl of 5×dilution buffer (400 mM Tris-HCl, 10 mM Mg2CL) in a total volume of 10 μl. The resulting sequences were then analyzed using the SeqMan program (DNASTAR, Lasergene). Distinct new MIB1 and MIB6 sequences detected at least twice in two different PCR reactions were deposited in GenBank under the accession numbers FM207965–FM207986 and FM209517–FM209524, respectively.

### Phylogenetic analyses

Phylogenetic analyses were performed upon 93 and 58 nucleotides of the MIB1 and MIB6 flanking sequence, respectively, applying the HKY+G substitution model inferred for both dataset using the software modeltest 3.7 [38]. The Bayesian analyses were performed using the software mrbayes [39], in which two Markov Chains were run on 10×10^6^ generations with a sampling of 100 generations each. A run of this length allowed the standard deviation of allelic frequencies to pass below 0.01 and the potential scale reduction factor (PSRF) to reach a value of 1, as suggested by the authors. The first 25,000 trees (25%) were discarded from the analysis as a burn-in.

## Results and Discussion

### Physical localization of B genes and MIB loci

**Figure 1:**
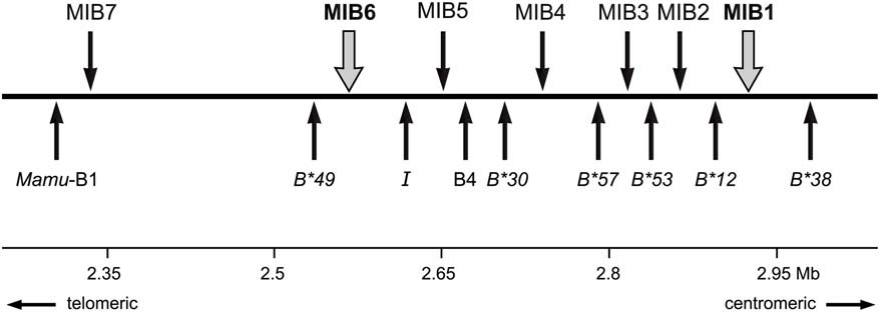
Partial physical map of the *Mamu-B* region with allocation of *B* genes and MIB loci. The names of the B1 to B9 locus designations (Daza-Vamenta et al. 2004) have been replaced by the latest *Mamu-B* loci/lineage names whenever possible (Otting et al. 2008).

*In silicio* analysis resulted in the identification of 12 MIB loci in a MHC heterozygous rhesus macaque using the NCBI Blast database (http://blast.ncbi.nlm.nih.gov/Blast.cgi) [29]. Seven MIB loci map to one haplotype, whereas the other five are located on the complementary chromosome, for which not all overlapping contigs are available [16], [21]. For that reason, attention was focused on the haplotype for which all relevant information is available. As can be seen, all seven MIB loci are situated next to *Mamu-B* (pseudo)genes, which correspond to the loci named B1 to B9, in their order of appearance as was defined by Daza-Vamenta and coworkers (Fig. 1, supplementary Table 1). By annotating the genes on the different BAC clones (supplementary Table 1) and by comparing them with the information in the NHP-MHC database, seven of the nine loci could be identified as expressed *Mamu-B* or *B*-like (*I*) genes [23], [24]. In the relevant cases, the internationally accepted names, as they appear in the NHP-MHC database, have been provided (Fig. 1). Loci B1 and B4, however, seem to represent pseudogenes or genes that are expressed on specialized tissues and/or at extremely low levels. Subsequent comparison confirmed that the seven expressed *B* genes are inherited together on the haplotype that in Indian animals is known as a B11 serotype [23].

**Table 1:** Mamu-B serotypes and correlating MIB6 and MIB1 genotype patterns

	B29	B24	B11	B13	B32	B2	B17	B26
**MIB6**	110 (111)	-	137, 140^a^ (137, 139^a^)	-	110 (111)	122 (123)	116 (117)	122 (123)
**MIB1**	148 (149)				148 (149)			
							154 (155)	
	(159)				158 (159)	157 (157)		160 (159)
						160 (161)		161 (161)
					165 (164)			
		168 (167)	166 (165)	168 (167)		168 (167)	170 (169)	
	166, 186 (165,183a)							166 (165)
	172 (171)							
								172 (173)
					174 (173)			
	176 (175)							
								181 (180)
	(183b)							
**Ani-mals**	2B, 2V, 2AF	2BX, 2AD, 98017	3C^a^, 96084	99029, 95014	2BZ, 94030, 3988, 95041,	2DE, 2DF	1R, 2AK, 2Z	99037, 9920

All animals are MHC homozygous and additionally derive from consanginous matings, except 96084 and 9220.

a This STR length is specific for the founder of animal 3C. STR lengths in brackets correspond to sequencing lengths (Fig 3 and 4). MIB1-STR lengths within a given row indicate that the MIB-surrrounding sequences are identical and cluster together (Fig. 3 and 4).

### MIB1 and MIB6 genotyping

Based on the genomic data, two primer pairs have been designed that should amplify specifically MIB1 and MIB6, respectively. As can be seen, in the case of the B11 serotype both MIB loci are situated between two expressed *Mamu-B* genes (Fig. 1). The two genes next to MIB1, Mamu-B*12 and -B*38, represent ‘majors’ and are transcribed at high levels. MIB6 maps between the Mamu-B*49 and *-I*, which was named B3 by other investigators [16]. The first one represents a ‘minor’ that is transcribed at low levels, whereas *Mamu-I* may represent a nonclassical [25].

MIB genotyping was then performed on a panel of MHC homozygous rhesus macaques, mostly of consanguineous origin, comprising eight different serotypes (Table 1). As expected, MIB1 and MIB6 genotyping of monkeys positive for the B11 serotype resulted in the definition of one single amplicon each. Amplification of MIB6 of the other B serotypes also resulted in one PCR product of distinct but variable length. Additionally, the Mamu-B11-specific STR displays length variation at the population level. The exception is provided by the B13 and B24 serotypes, for which no MIB6 locus could be amplified . In contrast, genotyping with the MIB1 primers resulted in far more complex patterns. The most parsimonious explanation is that the MIB1 primer pair, although specific for its locus on the B11 serotype, may amplify several related MIB loci in the case of other serotypes. Since the analysis was performed on truly homozygous animals, genotyping allows the characterization of one to five MIB1 amplicons per haplotype. The different amplification patterns of MIB6 as well as MIB1 appear, however, to be haplotype and serotype specific (Table 1).

**Figure 2:**
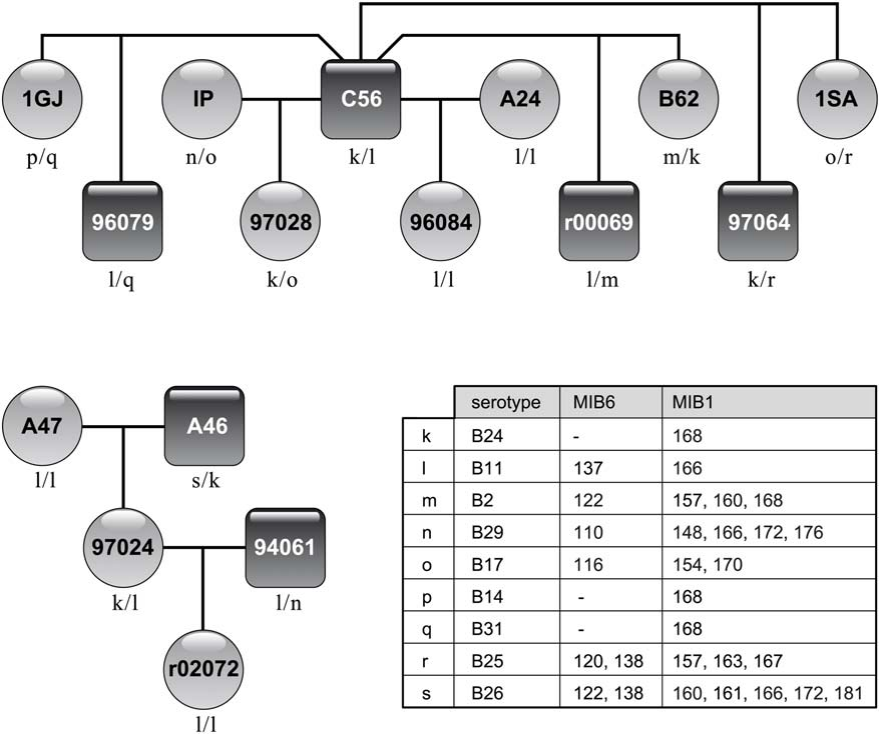
Pedigree of a macaque family (C56) showing the segregation of Mamu-B serotypes and MIB loci. Monkey A47 and his family has been added, since A47 is an MHC-identical sibling of A24. MIB1-183 of haplotype n, serotype B29, could not be detected although the haplotype is most probably identical to B29 of the homozygous monkeys (Table 1).

To verify that the different amplicons are inherited in a Mendelian manner, two families have been analyzed. Informative pedigree information of one family is shown, together with the corresponding serotypes as well as the MIB inheritance patterns (Fig. 2). The results verify that the STRs segregate according to Mendelian rules and illustrate that both microsatellite patterns are haplotype specific. Furthermore, this approach proves that the two STR lengths as defined for MIB6 of serotype B11 (Table 1) are not methodical artifacts but are founder haplotype specific (Fig. 2). Moreover, such an approach allows one to define the STR patterns belonging, for instance, to the B14, B25, and B31 serotypes, for which no homozygous typing cells are available. The main conclusion, however, is that genotyping with only two MIB microsatellites permits one to define most of the Mamu-B serotypes unambiguously. This may be of benefit of other researchers who are using rhesus macaques as nonhuman primate models to study human biology or disease. Discrimination at even higher levels can be achieved, as variation within a given serotype can be detected. An example is illustrated for the highly frequent serotype B26, which can be divided into at least two groups, one with one MIB6 amplicon, 122 (Table 1), and another group of monkeys with two MIB6 amplicons, 122 and 138 (Fig. 2). This suggests that gene content may differ even among identical serotypes, providing further evidence that this region is highly plastic.

### Diversity of MIB1 and MIB6 loci/alleles

**Figure 3:**
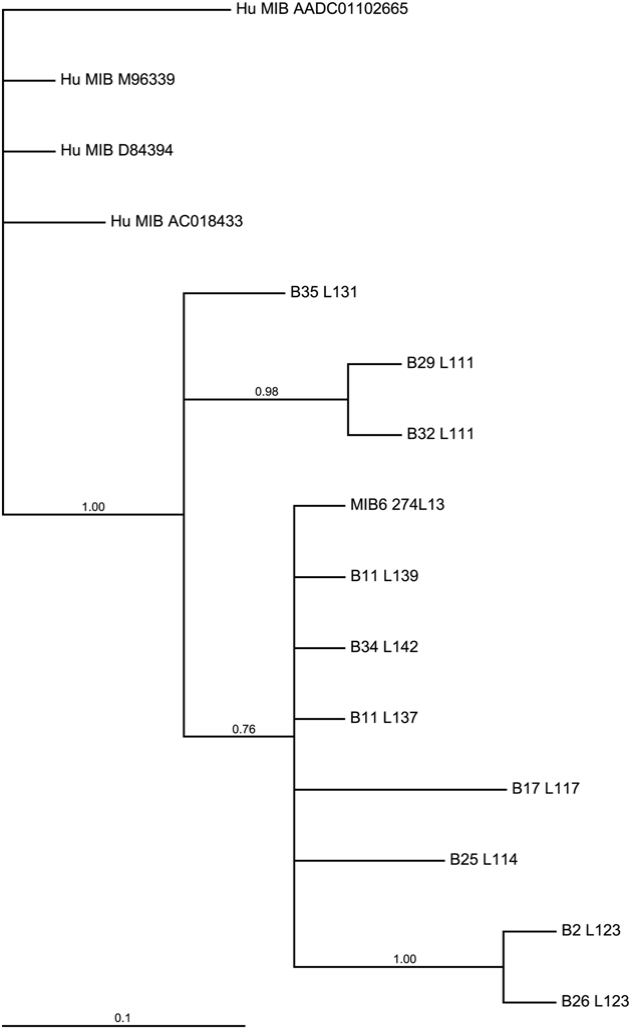
Phylogenetic tree of flanking sequences of MIB6 of humans and rhesus macaques. Numbers at nodes are posterior probability values for node support. Sequences are named by B serotype (B) and STR length (L).

To confirm that all amplicons indeed represent MIB loci, relevant PCR products obtained from the MHC homozygous animals studied have been cloned and sequenced. Additionally, MIB6 sequences of one B25 homozygous animal, and two MHC heterozygous animals, B34 and B35, for which the B haplotypes had been defined by segregation analysis, have been incorporated in the analysis (Fig. 3). All MIB6 amplicons detected by genotyping in MHC homozygous animals could be ascertained by sequencing. The MIB6 sequence of the B25 homozygous animal is different from the MIB6 STRs of the B25 serotype present in the macaque family (Fig. 2, 1SA and 97064), confirming the observation that variation within some of the serotypes can be discriminated by MIB genotyping. The MIB6 segments available for phylogenetic analysis, which excluded the repeat area but included informative mutations within the repeat, was too short to resolve phylogenetic relationships for all MIB sequences (Fig. 3). However, in the Bayesian analysis human MIB alleles clearly cluster apart from the rhesus macaque equivalents. Furthermore, the tree suggests the existence of deviating lineages such as the MIB6 of the B35 serotype but also of more recent duplications of MIB6 loci, as shown for serotypes B29 and B32. At this stage, it cannot be excluded that the latter represent allelic variants of one and the same locus. Furthermore, the tree confirms that the published MIB6 sequence [16] is identical to the MIB6 amplicon obtained from cells positive for the B11 serotype. (Fig. 3, Supplementary Table S1). The MIB6 sequence gained from the B34 serotype is also identical to the published sequence.

**Figure 4:**
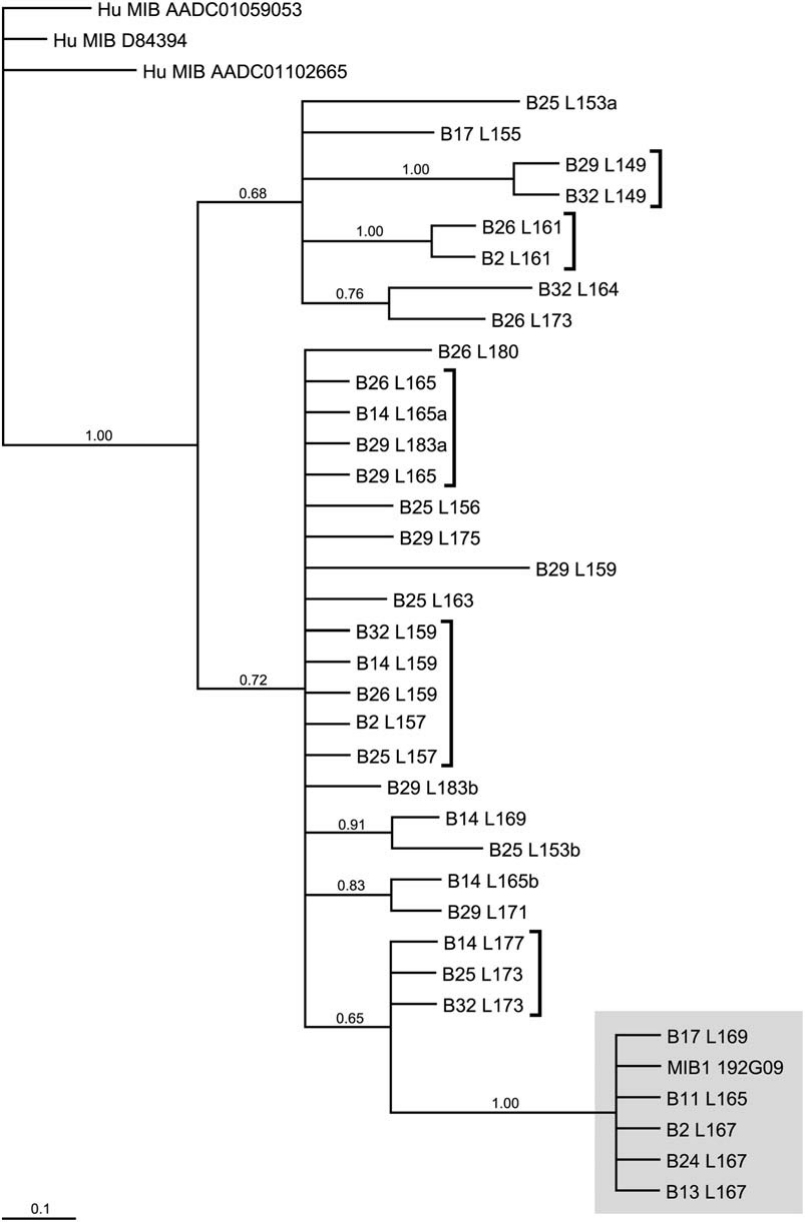
Phylogenetic tree of flanking sequences of MIB1 of humans and rhesus macaques. Numbers at nodes are posterior probability values for node support. Sequences are named by B serotype (B) and STR length (L). Braces or shadow indicate identical sequences differing only in STR lengths.

Phylogenetic analysis has also been performed for the MIB1 sequences that were obtained from the MHC homozygous animals (Fig. 4). As has been shown for MIB6, all MIB1 sequences determined by STR genotyping could be confirmed by sequencing. For serotype B29, one additional STR could be detected, MIB1-159, and two distinct STRs of the same length, 183a and 183 b, could be defined that were indistinguishable in STR genotyping (Fig. 4 and Table 1). Furthermore, this haplotype shows another peculiarity: namely, two STRs, 165 and 183a, that show identical repeat-surrounding sequences and therefore may represent a very recent duplication of the same MIB locus (Table 1 and Fig. 4). The MIB1 sequences of the B25 and B14 animals, respectively, are different from the MIB1 STRs detected in the same serotypes as present in the macaque family. This observation underscores the possibility to refine B serotypes by MIB typing. (Fig. 2, r (1SA and 97064) and p (1GJ); Fig. 4). In the phylogenetic tree, human MIB1 sequences cluster apart from the MIB1 STRs, as has been shown for MIB6. Again, the existence of deviating lineages as well as allelic sequences or recent duplications of MIB1 loci can be observed. Additionally, several groups of identical sequences are detected that indicate sharing of various MIB loci by different Mamu-B serotypes (Fig. 4, Table 1). The most deviating branch includes the MIB1 sequence of the published BAC clone 192G09 and the MIB1 locus of serotype B11 (shadowed). The identical STR segment is present on the B17, B24, B2, and B13 haplotypes with serotype-specific microsatellite lengths varying from 165 to 169 bp (Fig. 4, shadowed branch, and Table 1).

### MIB is a retroviral sequence closely linked to HERV16

**Figure 5:**
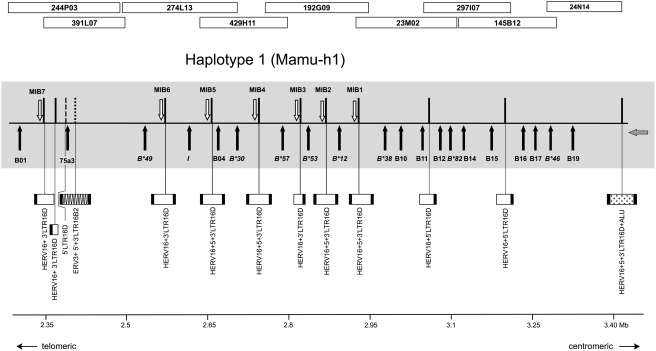
Physical map of the rhesus macaque *Mhc-B* region with allocation of all *Mamu-B* genes, MIB, loci and HERV16/HERV16-like sequences. Schematic drawing according to Daza-Vamenta et al. (2004) and *Mamu-B* loci/lineage designation according to Otting et al (2008). Arrow indicates transcription direction. Drawing is in scale, but HERV sequences are enlarged (10:1).

The observation that about half of the *Mamu-B* genes are coupled to MIB, whereas the others are not, persuaded us to have a more careful look, especially at the transposable elements which may be situated near the MIB loci. RepBase (http://www.girinst.org/censor/index.php) comparisons revealed that all published MIB sequences [16] are actually an integral part of an endogenous retrovirus (ERV3), called MTL2C2. This specific MLT2C2/MIB segment is always linked to a retroviral sequence of ∼5 kb, HERV16, generally accompanied by long terminal repeats (LTR) on both ends, designated 5′ and 3′LTR16D respectively (Supplementary Table S1, Fig. 5). Additionally, the *Mamu-B* loci and corresponding HERV16 segments are always separated by a similar distance of approximately 21 to 27 kb (Supplementary Table S1). The untranslated B4 gene not only lacks an adjacent MIB segment but also the corresponding HERV16 structure. B4 is situated much closer to its next *B* locus, B*30, than are the other telomeric *Mamu-B* genes are to each other. Therefore, it seems likely that the accompanying HERV16 and MLT2C2/MIB segment must have been lost during evolution. Again, the individual *B* loci of the telomeric section share approximately the same distances to each other, except that B1 and its next *B* locus, -B*49, are separated by a much longer stretch of DNA. In fact, a partial *B*-gene segment, named 75a3, comprising exon 1 to parts of exon 3, is located in this area. This partial *B* gene is surrounded by a truncated HERV16 segment and a 6.4 kb-long ERV3 retroviral sequence with 5′ and 3′LTRs that may have caused its truncation (Supplementary Table S1, Fig. 5). In contrast, the *B* genes of the centromeric part are situated much closer to each other than are the *B* genes of the telomeric part. Furthermore, no or only truncated HERV segments without 3′LTRs are localized between them. The most centromeric *B* locus, B19, actually has a HERV16 retroviral segment in the distant neighborhood, >90 kb apart, and its sequence is interspersed with *Alu* elements (Fig. 5).

The same situation can be observed for the partially mapped second haplotype [16], where MIB/HERV16 sequences are also localized next to transcribed *B* genes, whereas *B* pseudogenes do not show these endogenous retroviral sequences in their neighbourhood (Supplementary Table S1). Thus, it is conceivable that the MLT2C2/MIB sequences were lost together with the whole or parts of the HERV16 retroviral segments in the centromeric half of the *Mamu-B* region.

### Possible tandem duplications of Mamu-B and HERV16/MIB loci and subsequent recombinations

**Figure 6:**
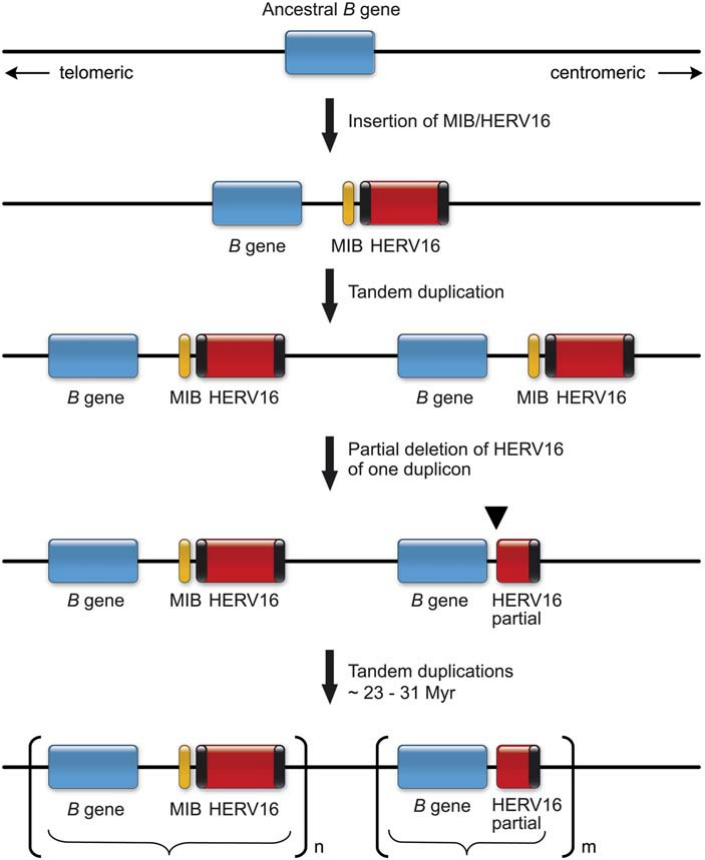
Hypothetical scheme of the evolution of the *Mamu-B* region by tandem duplication.

Twenty-eight duplicons have been described within the *Mamu-A* region (alpha block) and HERV16 sequences appear to map directly to their breakpoints [14]. For the *Mamu-B* region, a similar scenario may have been applicable (Fig. 6). The most likely scenario is that next to an ancestral *B* gene a long HERV16 sequence has been integrated accompanied by a MLT2C2 retroviral segment, which harbors the MIB microsatellite. The *B* gene-MIB/HERV16 tandem was then duplicated. One duplicon must have lost parts of its HERV16 segment together with the MIB marker, whereas the other duplicon remained intact. A similar step has already been suggested to have taken place before the evolution of the *HLA-B* region in humans [14], and based on these novel data it is likely to have occurred before the Old World monkey-hominoid split. In the ancestor of Old World monkeys, ∼25 Myr ago, both duplicons must have been subject to several rounds of tandem duplications (Fig. 6), a scenario that is supported by the phylogenetic analysis of different MIB loci in rhesus and cynomolgus macaques. This scenario may also help to shed light on the localization and functionality of *B* genes. An example is given by MIB1 (Fig. 1, Fig. 5), which, on this specific chromosome, is the most centromeric MIB locus followed upstream by *Mamu-B* loci with truncated or lost HERV16 sequences. The MIB1/HERV16 segment is located between the Mamu-B*12 and B*38 gene, and is characterized by high expression levels, the latter being the most centromerically situated *B* gene with characteristics of a major. Nearly all expressed *B* genes are localized on the telomeric, HERV16/MIB-associated part of the region, whereas the centromeric part harbors mostly unexpressed or infrequently expressed *B* loci (Fig. 5). The HERV16/MIB segment of the B*38 duplicon may have been the original that first became truncated in the past. The B*38 gene may have remained functional, since the truncation did not affect enhancer sequences. However, the 5′ enhancers and other regulatory sequences may have been affected during subsequent duplication steps. The centromeric *B*-genes themselves appear to be intact, which implies that they are perfect substrates to recruit new sequences for immune response purposes by means of recombination-like processes [40]. Furthermore, old sequences that once had a function in the immune response can be reactivated by recombination, positioning them in the correct context of functionally operative promotors and/or enhancer elements. The occurrence of recombination-like processes is supported by the observation that *Mamu-B* and MIB phylogenies are often incongruent. An example is given by the identical MIB1-STR segment that is present on B17, B24, B2, and B13 haplotypes (Table 1, rows; Fig. 4, shadowed). These B serotypes are all characterized by different combinations of known ‘major’ and ‘minor’ transcribed *B* alleles, which also cluster in different branches in the phylogenetic tree [24]. The only exception is the ‘major’ transcript B*3801 that is present on B2 as well as on some B11 serotypes. It cannot be excluded that these B serotypes share untranscribed *B* (pseudo)genes, since only transcripts have been described thus far [24]. However, the observation that the same MIB locus, MIB5, is present on both reported haplotypes [16], [29] but localized next to different *B* loci makes this possibility very unlikely. Thus, such sharing of identical MIB loci on different *B* haplotypes is most likely explained by recent crossing over events. It seems plausible that subsequent to the duplication processes, recombination has occurred between various MIB and *Mamu-B* loci, promoted by associated transposable elements acting as recombination hotspots [30]–[36], resulting in a hyperplastic *B* region. Extensive gene copy number variation may also be explained by unequal crossing over processes. Thus, microsatellite MIB typing and retrotransposon positioning, in addition to *B* loci characterization, is useful to decipher the compound functional and evolutionary genomics of the *Mhc* class I *B* region in Old World monkeys.

## Supporting Information

Table S1 Allocation of Mamu-B loci, MIB, and HERVs on the physical map of the rhesus macaque
(0.03 MB XLS)
